# An Update on the Lithogenic Mechanisms of Cholecystokinin a Receptor (CCKAR), an Important Gallstone Gene for *Lith13*

**DOI:** 10.3390/genes11121438

**Published:** 2020-11-29

**Authors:** Helen H. Wang, Piero Portincasa, Min Liu, Patrick Tso, David Q.-H. Wang

**Affiliations:** 1 Department of Medicine and Genetics, Division of Gastroenterology and Liver Diseases, Marion Bessin Liver Research Center, Einstein-Mount Sinai Diabetes Research Center, Albert Einstein College of Medicine, Bronx, NY 10461, USA; helen.jiang@einsteinmed.org; 2 Department of Biomedical Sciences and Human Oncology, Clinica Medica “A. Murri”, University of Bari “Aldo Moro” Medical School, 70124 Bari, Italy; piero.portincasa@uniba.it; 3 Department of Pathology and Laboratory Medicine, University of Cincinnati College of Medicine, Cincinnati, OH 45237, USA; lium@ucmail.uc.edu (M.L.); tsopp@ucmail.uc.edu (P.T.)

**Keywords:** bile salts, biliary sludge, cholesterol nucleation and crystallization, gallbladder motility, intestinal cholesterol absorption, *Lith* genes, lithogenic bile, microlithiasis, mucin gel

## Abstract

The cholecystokinin A receptor (CCKAR) is expressed predominantly in the gallbladder and small intestine in the digestive system, where it is responsible for CCK’s regulation of gallbladder and small intestinal motility. The effect of CCKAR on small intestinal transit is a physiological response for regulating intestinal cholesterol absorption. The *CCKAR* gene has been identified to be an important gallstone gene, *Lith13*, in inbred mice by a powerful quantitative trait locus analysis. Knockout of the *CCKAR* gene in mice enhances cholesterol cholelithogenesis by impairing gallbladder contraction and emptying, promoting cholesterol crystallization and crystal growth, and increasing intestinal cholesterol absorption. Clinical and epidemiological studies have demonstrated that several variants in the *CCKAR* gene are associated with increased prevalence of cholesterol cholelithiasis in humans. Dysfunctional gallbladder emptying in response to exogenously administered CCK-8 is often found in patients with cholesterol gallstones, and patients with pigment gallstones display an intermediate degree of gallbladder motility defect. Gallbladder hypomotility is also revealed in some subjects without gallstones under several conditions: pregnancy, total parenteral nutrition, celiac disease, oral contraceptives and conjugated estrogens, obesity, diabetes, the metabolic syndrome, and administration of CCKAR antagonists. The physical–chemical, genetic, and molecular studies of *Lith13* show that dysfunctional *CCKAR* enhances susceptibility to cholesterol gallstones through two primary mechanisms: impaired gallbladder emptying is a key risk factor for the development of gallbladder hypomotility, biliary sludge (the precursor of gallstones), and microlithiasis, as well as delayed small intestinal transit augments cholesterol absorption as a major source for the hepatic hypersecretion of biliary cholesterol and for the accumulation of excess cholesterol in the gallbladder wall that further worsens impaired gallbladder motor function. If these two defects in the gallbladder and small intestine could be prevented by the potent CCKAR agonists, the risk of developing cholesterol gallstones could be dramatically reduced.

## 1. Introduction

Based on accumulated evidence from epidemiological and clinical studies, as well as from animal and in vitro physical–chemical experiments, a critical concept on the pathophysiology and pathogenesis of cholesterol cholelithiasis has been proposed [1]. Five primary defects work together to enhance cholesterol cholelithogenesis, which include (i) *Lith* genes and genetic factors; (ii) hepatic hypersecretion of biliary cholesterol, inducing cholesterol-supersaturated gallbladder bile, i.e., high cholesterol saturation index (CSI); (iii) rapid cholesterol nucleation and crystallization and accelerated growth of solid cholesterol crystals; (iv) dysfunctional gallbladder motility, leading to impaired gallbladder emptying and refilling with mucin hypersecretion and gel formation, ultimately promoting the development of biliary sludge, i.e., the precursor of gallstones; (v) intestinal factors, including increased delivery of the cholesterol absorbed from the small intestine to the liver for biliary hypersecretion, alterations in gut microbiota, and sluggish intestinal transit. All of these five defects act together to dramatically reduce cholesterol solubility in bile, greatly promote cholesterol nucleation and crystallization, and accelerate the growth and agglomeration of solid plate-like cholesterol monohydrate crystals to form microlithiasis and eventually macroscopic gallstones [2,3,4,5,6].

The gallbladder volume is approximately 30 to 60 mL and is often changed, greatly depending on fasting or fed conditions [7]. Notably, the size of gallbladder becomes large after bile is concentrated and stored in the fasting state, whereas it is small following its postprandial emptying [8]. The gallbladder repeatedly contracts many times throughout the day. Depending on the intensity of neurohormonal response to fat and proteins in the diet, an effective postprandial gallbladder emptying often releases approximately 50% of bile into the proximal small intestine to enhance the digestion and absorption of triglycerides, cholesterol, and fat-soluble vitamins [9]. Moreover, between meals, hepatic bile is concentrated and stored in the gallbladder. As found by a combined cutting-edge technique using both cholescintigraphy and abdominal ultrasonography, the gallbladder commences emptying shortly after a meal, gradually reaches the lowest point in a varying time, and successively starts the refilling [10,11,12,13,14].

Many human and animal studies have found that defective gallbladder motility is intensely linked to gallstone formation [15] because it is often revealed in patients with cholesterol gallstones [16]. In addition, abnormal gallbladder motility is found in some gallstone-free subjects under several conditions such as pregnancy, total parenteral nutrition (TPN), celiac disease, oral contraceptives and conjugated estrogens, obesity, diabetes, and the metabolic syndrome [17,18,19,20,21,22,23,24]. Impaired gallbladder motor function could represent a pathophysiologically relevant stimulus predisposing to the formation of cholesterol gallstones [25].

It is well known that cholecystokinin (CCK) is a key neuro-intestinal peptide hormone and is synthesized and secreted by the enteroendocrine I cells that are located mainly in the proximal small intestine [26]. The concentration of CCK in the circulation is dramatically surged soon after consumption of a meal enriched with fat and proteins [27,28]. CCK plays a critical regulatory role, through both the CCK A receptor (CCKAR) and the CCK B receptor (CCKBR) pathways, in gallbladder smooth muscle and pancreatic acini, as well as at multiple levels in different organs such as the gastrointestinal tract, the enteric nervous system, and the brain. Furthermore, numerous clinical and pathophysiological studies have found that both CCK and CCKAR have a striking impact on the pathogenesis of cholesterol gallstone disease not only in mice, but also in humans. More importantly, the *CCKAR* gene has been identified to be a critical gallstone gene, called *Lith13*, in inbred strains of mice by a powerful genetic technique, the quantitative trait locus (QTL) analysis [1,29,30,31]. Furthermore, epidemiological investigations and clinical studies have found that the *CCKAR* gene is an important gallstone gene that is associated with increased prevalence of cholesterol gallstone disease in humans [32,33,34]. In this review article, we summarize the latest progress in the discovery of *Lith13* and its genetic analysis in mice and humans, as well as the lithogenic mechanisms of *Lith13* at a cellular and molecular level and its critical role in pathogenesis of cholesterol gallstone disease not only in mice, but also in humans.

## 2. Identification of *Lith13* in Mice

In 1928, a new protein named CCK was first identified from the small intestinal extracts, and subsequently, it was found that CCK is a hormone that can cause a dramatic gallbladder contraction and emptying [35]. The mRNA sequence of CCK includes 750 bases, with 345 being protein coding. The preproCCK, a translational product, has 115 amino acid residues. The bioactive CCK peptides are derived from the subsequent 58 amino acid residues (CCK-58), and the species variation is minor in this sequence [36]. Subsequent biochemical and physical–chemical studies have found that CCK plays a key role in regulating gallbladder motility, which is involved in the digestion and absorption of intestinal triglycerides, cholesterol, and fat-soluble vitamins. Based on molecular biological and genetic studies, the *CCK* gene is located on chromosome 3 (p22.1) in humans, chromosome 9 (72.43 cM) in mice, chromosome 8 (q32) in rats, chromosome 23 in dogs, chromosome 2 in chickens, chromosome 22 in cattle, and chromosome 3 in chimpanzees.

In 1992, the *CCKAR* gene was first cloned from the rat pancreas [37], which consists of five exons interrupted by four introns in all species studied. When estimated from the transcription start site to the poly a signal, the human *CCKAR* gene is 11.0 kb in length. Furthermore, the *CCKAR* gene in mice and the *CCKAR* gene in rats are roughly 9.0 kb and 9.5 kb in length, respectively. The *CCKAR* gene is found to be located on chromosome 4 (p15.2) in humans, chromosome 5 (29.52 cM) in mice, chromosome 14 (q11) in rats, chromosome 3 in dogs, chromosome 4 in chickens, chromosome 6 in cattle, and chromosome 4 in chimpanzees.

In the same year after the *CCKAR* gene was found, a new CCK receptor was identified in which a functional gastrin receptor, named CCKBR, was cloned from a canine parietal cell expression library [38]. Resembling CCKAR, CCKBR has seven hydrophobic segments that represent transmembrane helices and form a helical bundle domain and is typical of Family A in sharing the signature sequences of this family within these regions. However, CCKBR displays a different physiological function compared to CCKAR. The *CCKBR* gene encodes a G protein-coupled receptor for CCK and gastrin, regulatory peptides of the brain and gastrointestinal tract. The CCKBR is a type B gastrin receptor, which has a high affinity for both sulfated and nonsulfated CCK analogs and is found principally in the central nervous system and the gastrointestinal tract. On the basis of molecular biological and genetic investigations, the *CCKBR* gene is found to be located on chromosome 11 (p15.4) in humans, chromosome 7 (55.86 cM) in mice, chromosome 1 (q33) in rats, chromosome 1 in chickens, chromosome 15 in cattle, and chromosome 11 in chimpanzees.

Subsequently, the genomic structures of *CCKAR* and *CCKBR* were carefully compared among mice, rats, and humans. The physiological roles, biochemical functions, and biological activities of CCK, CCKAR, and CCKBR are comprehensively investigated, with a focus on gallbladder contractility, gastrointestinal transit, digestion and absorption of intestinal lipids and nutrients, the enterohepatic circulation of bile salts, glucose and energy metabolism, and neurotransmission in the brain [39,40,41,42,43,44]. In addition, clinical studies have found that CCK, CCKAR, and CCKBR are involved in many metabolic disorders such as cholesterol gallstone disease, obesity, diabetes, and the metabolic syndrome, as well as some gastrointestinal tract disorders, brain diseases, and cancers in the digestive system [36].

A principal goal of QTL analysis is to help researchers explore the possibility of whether phenotypic differences are predominantly due to a few loci with major effects, or to many loci, each with minor effects. Moreover, QTL genetic analysis is a statistical method that can link certain complex phenotypes to specific regions of chromosomes. QTL technique is particularly helpful in bridging the gap between lithogenic (*Lith*) genes and the phenotypic traits (gallstone formation) that result from the *Lith* genes. It is a powerful method to explain the genetic basis of variation in complex traits such as cholesterol gallstone disease. Similar to humans, it has been found that there is a striking difference in gallstone prevalence among various strains of inbred mice fed a lithogenic diet. Using QTL mapping methods, genetic studies have been performed to investigate *Lith* genes in different strains of inbred mice fed the lithogenic diet for 8–12 weeks [45]. For example, C57L/J mice susceptible to gallstone formation, whereas AKR/J mice are resistant; in both strains, gallbladder bile is supersaturated with cholesterol but to different degrees. Based on QTL analysis, at least two gallstone QTL regions have been identified in two different chromosomes, i.e., *Lith1* and *Lith2* QTL regions on chromosomes 2 and 19 in C57L/J mice.

**Figure 1:**
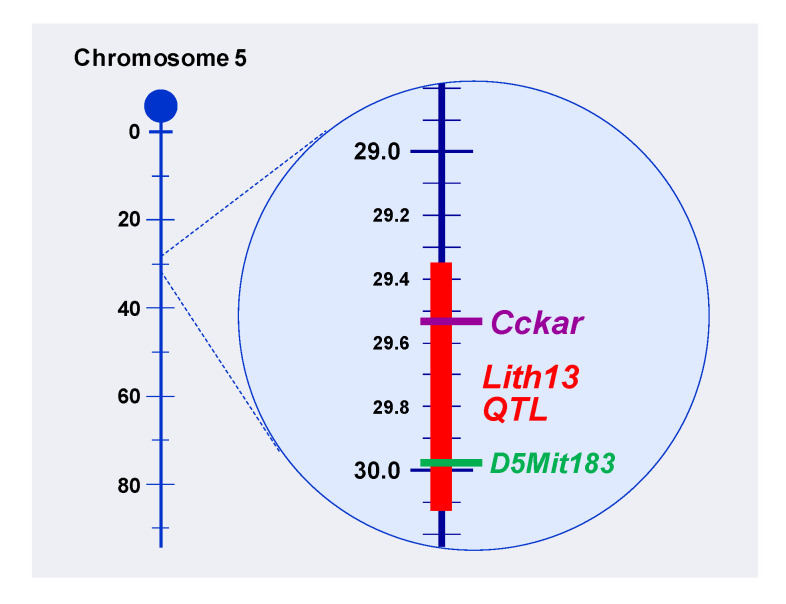
Composite map of quantitative trait locus (QTL) for the *Lith13* gene that is mapped to mouse chromosome 5. A vertical line is shown for chromosome 5, with the centromere being at the top; genetic distances from the centromere (horizontal white lines) are listed to the left of the chromosomes in centimorgans (cM). Chromosomes are drawn to scale, based on the estimated cM position of the most distally mapped locus taken from Mouse Genome Database. The *Lith13* QTL is represented by a vertical red line and the location of the cholecystokinin A receptor (*CCKAR*) gene is showed by a horizontal purple line. A genetic biomarker, *D5Mit183,* which is co-localized with *Lith13,* is signposted by a horizontal green line with the marker symbol to the right.

Using the same genetic approaches, a comprehensive investigation on the difference in gallstone prevalence between 129S1/J and CAST/J mice was carried out. As shown in Figure 1, *Lith13* is mapped to mouse chromosome 5 in inbred strains of 129S1/J mice, as analyzed by the QTL analysis. Notably, *Lith13* is co-localized with a genetic biomarker *D5Mit183* at roughly 30 centimorgans (cM), and within the *Lith13* QTL region, the *CCKAR* gene is a powerful candidate for this gallstone gene. Furthermore, genotyping and phenotyping studies have found that *CCKAR* is a key gallstone gene, *Lith13*, in mice [1,29,30,31].

## 3. Regulation of Gallbladder and Gastrointestinal Motility, as well as Pancreatic Secretion by CCK and CCKAR

It is well known that among the nutritional components, digested fat, as well as proteins and L-amino acids are the most important triggers to stimulate CCK release from the enteroendocrine I cells, whereas carbohydrates induce only a low level of the secreted CCK [46]. After CCK is secreted by the intestine, it enters the circulatory system. Subsequently, CCK triggers the secretion of digestive enzymes by the gastrointestinal tract and pancreas, as well as the release of bile from the gallbladder, respectively [26,47,48,49,50,51,52]. CCK consists of a group of variable numbers of amino acids, which depends on post-translational conversion of the protein product (i.e., preproCCK) of the *CCK* gene. Therefore, CCK in plasma is a family of hormones that are identified by the number of amino acids, including CCK-58, CCK-33, CCK-22, and CCK-8. In humans, CCK-33 and CCK-22 are the major components in circulation, whereas CCK-58 and CCK-8 are also present in plasma [53]. As both CCK-33 and CCK-8 display the same cholecystokinetic and pancreozymic potency on a molar base, what the enteroendocrine I cells release during meals may be less vital. However, the clearance rate of CCK-8 from the circulation is much faster compared with that of CCK-58, CCK-33, and CCK-22 [54]. Most of the CCK peptides have a sulfate group attached to the tyrosine in position 7 in the C-terminus. Such a chemical structure is essential for the ability of CCK to activate CCKAR. Although nonsulfated CCK peptides also exist, it cannot activate CCKAR.

Notably, CCK plays a critical role through the CCKAR signaling cascade in regulating a variety of physiological processes, including gallbladder contraction, sphincter of Oddi relaxation, increased bile flow, and accelerated small intestinal transit, as well as stimulation of pancreatic secretion, inhibition of gastric emptying and acid secretion, relaxation of lower esophageal sphincter tone, slowing of colonic motility, and regulation of satiety [26]. These are interceded by CCKAR mainly on gallbladder smooth muscle, pancreatic neurons in humans, and also directly on pancreatic acinar cells in rodents, as well as on pyloric smooth muscle, enteric neurons, and nuclei in the central nervous system [26]. By contrast, CCKBR is present largely in the gastric oxyntic mucosa and widely distributed in the brain, with its highest expression levels being in the striatum, cerebral cortex, and limbic system [55]. In addition to stimulating gastric acid secretion, CCKBR is likely to have an effect on anxiety and nociception. As this chapter is focused on the lithogenic mechanisms of *Lith13* on cholesterol gallstone formation, we will discuss the critical role of CCK in regulating gallbladder motility and gastrointestinal transit, as well as pancreatic secretion via the CCKAR cascade.

The secretion of CCK by the enteroendocrine I cells is stimulated primarily by chyme enriched with fat and/or proteins after these semifluid masses of partly digested food are expelled by the stomach into the duodenum [56]. Under normal physiological conditions, plasma CCK concentrations are very low (<1 pmol/L) compared with other pancreatic and gastrointestinal hormones [57]. During maximal stimulation, plasma CCK concentrations increase to 3–5 pmol/L in 20 min and subsequently diminish gradually to a basal level [57]. Notably, the concentration of CCK in the circulation is able to trigger gallbladder contraction for release of bile to the duodenum, as well as secretion of pancreatic juice and enzymes during meals, which greatly promote the digestion and absorption of intestinal nutrients such as fat, proteins, and carbohydrates, as well as cholesterol and fat-soluble vitamins [58]. As CCKAR is expressed predominantly in the gallbladder, sphincter of Oddi, pancreas, small intestine, gastric mucosa, and pyloric sphincter, CCK can exert its physiological functions via the CCKAR signaling pathway for regulating diverse digestive processes such as gallbladder contraction for release of bile to the duodenum, and secretion of pancreatic juice and enzymes, as well as small intestinal transit, and gastric emptying [36].

**Figure 2:**
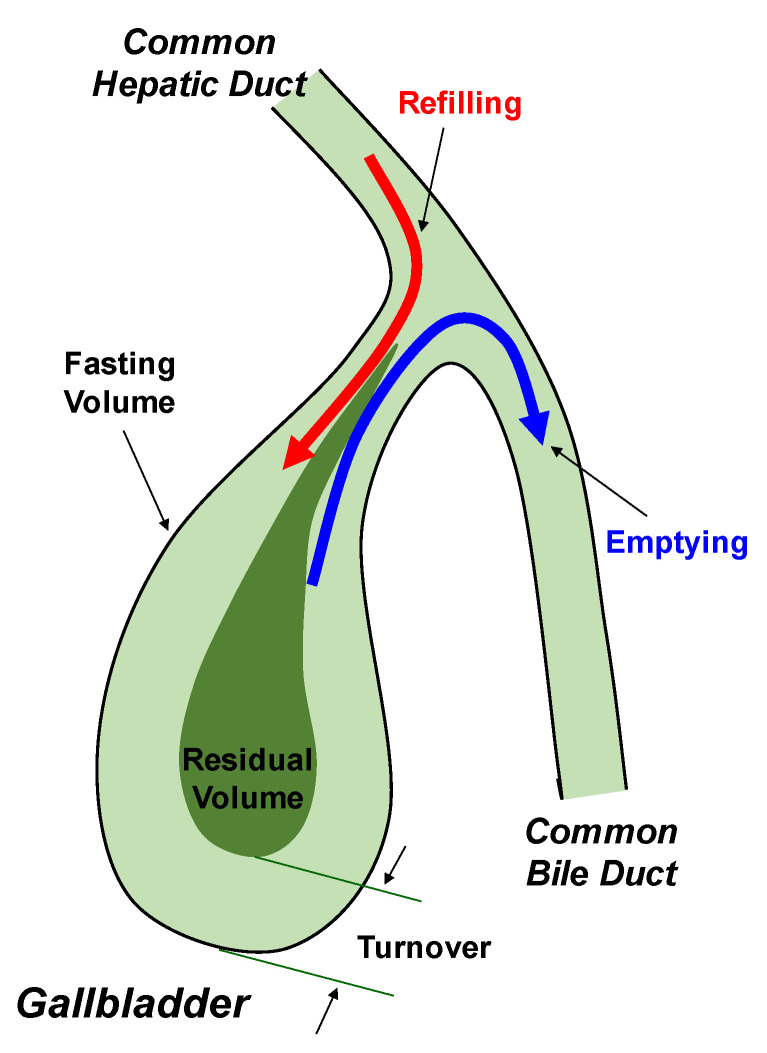
Shows a working model for gallbladder motor function. Arrows in (red) and out (blue) of the gallbladder represent postprandial gallbladder refilling and emptying. The difference between the fasting and the residual gallbladder volumes represents bile turnover. See text for further description.

The gallbladder is a major organ for the storage of bile secreted by the liver and its size varies noticeably. The gallbladder is large in size after hepatic bile is concentrated and stored during the fasting period, whereas it is small in size after its emptying over the postprandial period [8]. Between meals, hepatic bile at a continuous flow enters the gallbladder for storage, where bile is greatly concentrated by 3-fold to 10-fold. Notably, bile salts are essential for the digestion and absorption of triglycerides, cholesterol, and fat-soluble vitamins in the small intestine [59]. Bile salts are synthesized by the liver through the conversion of cholesterol and they are conjugated with taurine and glycine in a ratio of 1:3 in humans [60,61,62]. The liver secretes bile salts into hepatic bile that continuously enters the gallbladder through the biliary tract. After hepatic bile is concentrated by the gallbladder, the concentration of bile salts is increased dramatically, which greatly facilitates the bile storage in the gallbladder [7]. When food enriched with fat and protein enters the duodenum and stimulates CCK secretion, CCK triggers gallbladder emptying by activating CCKAR on the smooth muscles of gallbladder with a potency correlated to the concentration of sulfated CCK-8 in plasma. Consequently, gallbladder bile is largely emptied into the small intestine via CCK-mediated rhythmic contraction and relaxation of smooth muscles of the common bile duct and the sphincter of Oddi. Overall, the gallbladder releases bile to the proximal small intestine where biliary bile salts emulsify dietary fat and promote the digestion and absorption of cholesterol, fatty acids, and fat-soluble vitamins [9]. Figure 2 exhibits a working model for gallbladder emptying, refilling, and bile turnover.

Notably, CCK also plays a key role through the CCKAR cascade in the regulation of the gastrointestinal tract motility. However, there is a species difference in the impact of CCK on gastric emptying. CCK impedes gastric motility and food intake, thereby delaying transfer of food to the duodenum likely through activation of vagal afferent neurons in which there is a striking express of CCKAR. Furthermore, gastric emptying is hampered possibly via stimulation of vagovagal reflexes. Consequently, this causes relaxation of the gastric corpus and increases resistance to chyme flow across the pyloric sphincter of stomach. It has been found that deletion of either the *Cck* or the *CCKAR* gene in mice leads to a dramatically sluggish small intestinal transit [31,63]. This strongly indicates that CCK can accelerate the transit rate of small intestine via the CCKAR pathway. Furthermore, accumulated evidence from human and animal studies has shown that delayed small intestinal transit significantly increases intestinal cholesterol absorption [31,63,64]. This indicates that CCK and CCKAR can work together on modulating the absorption efficiency of intestinal lipids.

In addition, CCK stimulates the secretion of many pancreatic enzymes such as pancreatic amylase, trypsinogen, and chymotrypsinogen, as well as of some small intestinal enzymes including alkaline phosphatase, enterokinase, and disaccharidase [65]. Of special note, although CCK plays a key role in promoting pancreatic exocrine secretion, there is a species difference in the regulatory mechanisms. CCK triggers the secretion of pancreatic enzymes through the cholinergic route in humans, whereas it is not found in rodents [66,67,68]. Moreover, CCK could activate bicarbonate secretion by the ductular cells in the liver.

## 4. Role of *Lith13* in Impairing Gallbladder Motility

As monitored by ultrasonography, clinical studies have observed that impaired gallbladder emptying could precede the formation of gallstones [69]. Increased gallbladder size in the fasting state and sluggish gallbladder motility, as well as an enlarged residual volume after gallbladder emptying are often found in patients with cholesterol gallstones, regardless of stone sizes or bile lithogenicity [6]. Moreover, abnormalities in gallbladder contractility in response to exogenously administered CCK-8 are revealed mostly in cholesterol gallstone patients [70]. Further studies reveal that gallbladder inflammation is often insignificant in these patients. In addition, incomplete gallbladder emptying is present in a subgroup of patients with pigment gallstones. These patients often display a mild degree of impaired gallbladder emptying in the lack of a large gallbladder size under fasting conditions and do not show inflammation in the gallbladder [71].

More interestingly, defective gallbladder motor function is often uncovered in pregnant women, postmenopausal women receiving estrogen replacement therapy, and women taking oral contraceptives [72,73,74,75]. Augmented fasting gallbladder size and sluggish gallbladder motility also exist in some gallstone-free subjects under certain conditions, as discussed above [17,18,19,20,21,22,23,24]. All these individuals with dysfunctional gallbladder contractility predispose to gallstone formation [12,25].

Notably, impaired gallbladder contraction and emptying in cholesterol gallstone patients is not attributable to the physical presence of gallstones per se in the gallbladder lumen because defective gallbladder motility is not associated with the number or size of gallstones, and lithotripsy-induced gallstone ablation cannot restore impaired gallbladder contractility [76]. Gallbladder emptying in response to exogenously administrated CCK-8 is dampened in patients with cholesterol gallstones compared to healthy control subjects [77], indicating that dysfunctional CCKAR in the gallbladder could be attributable to diminished expression of CCKAR, or binding capacity of CCK to CCKAR, or both, in these patients.

As aforementioned, the *CCKAR* gene has been revealed to be a mouse gallstone gene, *Lith13*. As CCK and CCKAR work together to impact gallbladder emptying, it has been extensively studied how the regulatory functions of the CCK and CCKAR pathway are impaired. Due to sluggish contractility of gallbladder smooth muscles in gallstone patients and animal models of cholesterol gallstones, it is likely that the contractile mechanisms of gallbladder involve activation of phospholipase C by the CCKAR signaling pathway, leading to signal-transduction decoupling when cholesterol is largely accumulated in the sarcolemmal membrane of smooth muscles in the gallbladder [78,79,80,81,82,83,84]. This could be attributable to a striking decrease in expression of the *CCKAR* gene or its dysfunction in the smooth muscles of gallbladder. This may lead to an impairment in gallbladder contractility, which is likely induced by excess cholesterol accumulation in the caveolin rafts of sarcolemma of smooth muscles. Moreover, the CCKAR signaling pathway that regulates gallbladder emptying could be worsened by the cholesterol absorbed from supersaturated gallbladder bile, as found in CCKAR knockout mice. This would further enhance cholelithogenesis [85,86,87].

Based on the results from in vitro studies, abnormalities in the binding capacity of CCK-8 to CCKAR on the membrane of gallbladder smooth muscles are uncovered in patients with cholesterol gallstones compared with healthy subjects, showing reduced contractility of isolated gallbladder smooth muscle cells and impaired contraction of isolated gallbladder smooth muscle strips and whole gallbladder preparations [88,89,90,91]. Interestingly, defective gallbladder motility associated with cholesterol gallstones is reversible at an early stage and could be attributed to a removal of excess cholesterol that is accumulated in the membrane of smooth muscles of gallbladder [92]. This implies why gallbladder contraction and emptying is markedly reduced under conditions of bile supersaturation with cholesterol and before gallstone formation [93,94,95]. Additionally, it is likely that the intracellular functions of smooth muscle contraction could be intact in the gallbladder of patients with cholesterol gallstones. This implies that there may be an increased gallbladder cholesterol absorption from supersaturated bile, leading to a reduction in contractile function of smooth muscles in the gallbladder [96]. These changes could cause stiffening of sarcoplasmic membrane and this abnormality could be secondary to a surge in the concentration of cholesterol that is accumulated in the membrane of smooth muscles of gallbladder [97,98,99]. As a result, even though CCK-8 binds to CCKAR on the smooth muscles of gallbladder, G proteins cannot be activated and gallbladder contraction and emptying are diminished under lithogenic conditions [100,101,102,103].

Notably, the fate of excess amounts of cholesterol that is absorbed by the epithelial cells of gallbladder could be the same as the events for the formation of an atherosclerotic plaque in the artery. Cholesterol could be persistently absorbed by the epithelial cells from supersaturated gallbladder bile. Subsequently, the unesterified cholesterol largely and rapidly diffuses to the muscularis propria because of the lack of an intervening muscularis mucosae and submucosa in the gallbladder. As lipoproteins cannot be synthesized by the gallbladder for pumping out excess cholesterol to plasma, these excess amounts of cholesterol are removed from the smooth muscles and mucosa of gallbladder either by esterification for storage or by diffusion back into bile. As gallbladder bile is continuously increased with cholesterol in the lithogenic state, the pathway for cholesterol diffusion back into bile is impeded [104]. As a result, most, but not all, cholesterol could be esterified by the acyl-coenzyme A: cholesterol acyltransferase (ACAT) of gallbladder [105]. Similar to an atherosclerotic plaque, the cell membrane of mucosa and smooth muscles is enriched with cholesterol, leading to the accumulation of large amounts of cholesteryl esters. Additionally, the cholesterol molecules may be assimilated in the membrane bilayer of smooth muscles of gallbladder, thereby leading to a great change in the physical state of phospholipid molecules, as shown by their intensified rigidity [106,107,108]. As a result, there is a striking reduction in gallbladder contraction and emptying because the CCKAR signaling cascade activated by CCK-8 is impaired. Moreover, excess amounts of cholesterol that is absorbed from supersaturated bile could trigger inflammation and proliferation in the mucosa and lamina propria of gallbladder [109,110,111].

## 5. Effect of *Lith13* on Delaying Intestinal Transit Time

As high intestinal cholesterol absorption efficiency is associated with increased gallstone prevalence in inbred mice, this suggests that high cholesterol diet and high intestinal cholesterol absorption could be two independent risk factors for the formation of cholesterol gallstones [112]. In addition, C57L/J mice, an gallstone-susceptible strain, display significantly higher intestinal cholesterol absorption efficiency compared to AKR/J mice, a gallstone-resistant strain [113,114,115]. Thus, there may be a striking difference in the hepatic metabolism of chylomicron remnant cholesterol between C57L/J and AKR/J mice, which may explain why cholesterol-supersaturated gallbladder bile is formed much earlier in the former than in the latter. As a result, large amounts of the cholesterol absorbed from the small intestine are secreted into bile, leading to hepatic hypersecretion of biliary cholesterol in C57L/J mice under conditions of feeding a lithogenic diet [112]. Notably, it is still unclear whether high intestinal cholesterol absorption efficiency is an independent risk factor for cholesterol gallstone disease in humans [116].

More interestingly, it has been observed that delayed large intestinal transit is associated with increased glycodeoxycholate and taurodeoxycholate concentrations in bile of some gallstone patients [117]. As shown in human and mouse studies, it is highly likely that sluggish intestinal transit may play a causal role in the formation of high deoxycholate levels and bile lithogenicity [118]. Clinical studies have revealed that there is a positive relationship between a sluggish colonic motility, high biliary glycodeoxycholate and taurodeoxycholate concentrations, and rapid cholesterol crystallization in octreotide-treated patients with acromegaly, promoting the formation of cholesterol gallstones [119]. More importantly, there is a correlation among increased deoxycholate concentrations in bile, augmented numbers of Gram-positive anaerobic bacteria, and higher 7α-dehydroxylase activity in the cecum of gallstone patients compared to healthy controls [120,121,122]. By contrast, antibiotic treatment in these patients could inhibit activity of intestinal 7α-dehydroxylase and consequently reduce glycodeoxycholate and taurodeoxycholate levels and cholesterol concentrations in bile. Of note, biliary taurodeoxycholate concentrations are significantly higher in C57L/J mice compared to AKR/J mice. As a result, there is more rapid cholesterol supersaturation, cholesterol crystallization, and gallstone formation in the former than in the latter [123,124,125].

As the *CCKAR* gene is also expressed in the smooth muscles of small intestine, CCKAR is involved in the regulation of small intestinal motility. Small intestinal transit time is significantly slower in CCKAR knockout mice than in wild-type mice, irrespective of whether the lithogenic diet or the regular chow diet is fed [31]. Furthermore, sluggish small intestinal transit enhances intestinal cholesterol absorption because the residence time of the sterols in the lumen of small intestine is extended [64]. This could amplify cholesterol’s assimilation with mixed micelles in the intestinal lumen and promote partitioning of cholesterol molecules from mixed micelles for capture by Niemann-Pick C1-like 1 (NPC1L1), an intestinal cholesterol transporter on the apical membrane of enterocytes [126]. This suggests that the transit rate of cholesterol in the entire small intestine is also a critical factor for the regulation of cholesterol absorption. The results from a highly precise cholesterol balance study show that intestinal cholesterol absorption efficiency is significantly higher in CCKAR knockout mice than in wild-type mice, regardless of whether the lithogenic diet or the regular chow diet is fed [31]. Additionally, large amounts of the cholesterol of intestinal origin are transported to the liver by chylomicron remnants, which further enhances hepatic hypersecretion of biliary cholesterol and promotes the formation of cholesterol-supersaturated bile [112].

The transit time of the small and large intestines of patients with cholesterol gallstones has been found to be prolonged [127]. Notably, delayed transit time of the large intestine, together with greater activity of 7α-dehydroxylase by gut anaerobes, dramatically increases the synthesis and absorption of deoxycholate from the large intestine [122,128]. However, these findings on delayed large intestinal motility could not explain the mechanism of why intestinal cholesterol absorption is increased. Notably, increased levels of glycine- and taurine-conjugated deoxycholate produce a lithogenic effect on promoting hepatic hypersecretion of biliary cholesterol and accelerating cholesterol nucleation and crystallization in gallbladder bile [121,129,130,131]. However, such results are different between humans and mice. This may be attributable to a difference in bile salt metabolism. For example, feeding 0.5% deoxycholic acid increases taurodeoxycholate in the bile salt pools to 27% from 3.4% on the regular chow diet in male C57L/J mice, but small intestinal transit time is still unchanged [114].

## 6. Lithogenic Mechanisms Underlying the Role of *Lith13* in Cholesterol Crystallization and Gallstone Formation in Mice

Deletion of the *CCKAR* gene in mice leads to dysfunctional muscle contractility in the gallbladder and small intestine, thus impairing gallbladder contraction and delaying small intestinal motility [31]. Consequently, intestinal cholesterol absorption is dramatically augmented in CCKAR knockout mice, which, in turn, promotes biliary cholesterol hypersecretion. Moreover, dysfunctional CCKAR also enlarges gallbladder volume and promotes gallbladder cholesterol absorption from saturated bile, further worsening gallbladder contractility and leading to gallbladder stasis. Prolonged small intestinal transit rate contributes to the formation of cholesterol gallstones by enabling more efficient cholesterol absorption by the enterocytes and promoting hepatic cholesterol secretion, followed by rapid cholesterol crystallization, as well as solid crystal growth and agglomeration in a “large lax and lazy” gallbladder [4]. Furthermore, the potent CCKAR antagonist, devazepide, greatly enhances cholesterol cholelithogenesis by dramatically reducing gallbladder contraction and emptying, delaying small intestinal transit, disrupting biliary cholesterol metabolism, and augmenting intestinal cholesterol absorption in mice [132]. Overall, these results strongly imply that gallbladder hypomotility, delayed small intestinal motility, and amplified intestinal cholesterol absorption are all critical risk factors for the formation of cholesterol gallstones in CCKAR knockout mice fed the lithogenic diet.

In the lithogenic state, the evolutionary sequences of gallstone formation are characterized by the initial accumulation of mucin gel, followed by the appearances of liquid crystals and/or anhydrous cholesterol crystals and classical plate-like cholesterol monohydrate crystals, and then agglomerated cholesterol crystals, sandy stones, and gallstones in CCK knockout mice fed the lithogenic diet for 8 weeks [133,134]. These findings are consistent with the results found in other animal models of cholesterol gallstones, e.g., CCKAR knockout mice [31], C57L/J mice [123], and prairie dogs [135,136]. It is well known that there is a crystallization sequence from liquid crystals to classical plate-like cholesterol monohydrate crystals in human and mouse gallbladder bile [137,138,139]. Furthermore, the evolution from anhydrous cholesterol crystals to solid cholesterol monohydrate crystals [123,140,141] is important in the early stage of cholesterol gallstone formation in CCK knockout mice with dysfunctional gallbladder motility. Thus, these two cholesterol crystallization pathways play a critical role in gallstone formation in these mice. These cholesterol crystallization pathways in mice are the same to those identified in native human bile and in model bile for physiological lipid composition [123,140,141,142]. Notably, there is more rapid evolution from solid cholesterol crystals to gallstones in CCK knockout mice than in other mouse models of cholesterol gallstones on the same lithogenic diet [123], which highlights the importance of impaired gallbladder emptying. In addition to the same gallbladder phenotype, i.e., impaired gallbladder emptying in both CCK and CCKAR knockout mice, the identical evolutionary sequences from solid cholesterol crystals to gallstone formation are found in both strains of knockout mice.

Although over the past decades the growth habits of solid cholesterol crystals have been extensively investigated mainly in model bile systems for physiological lipid composition [143,144], it remains unknown whether these events occur in humans or mouse gallbladder bile. Interestingly, it has been revealed that there are three forms of the growth habits of solid cholesterol crystals in the early stage of gallstone formation in supersaturated gallbladder bile of mice fed the lithogenic diet, with all three modes increasing the size of solid cholesterol crystals and promoting crystal growth [133]. These results are in line with the sequences found in model bile systems [143]. Of special note is that when the values of CSI in lithogenic bile are high, both the spiral dislocation growth and the twin crystal growth pattern are major crystal growth habits [143]. When the values of CSI are low, the third mode of crystal growth habit, e.g., proportional enlargement pattern, is found. Obviously, dysfunctional gallbladder motility could increase the size of solid cholesterol monohydrate crystals because of a more rapid growth of cholesterol crystals through these three crystal growth patterns in CCK knockout mice compared to wild-type mice.

**Figure 3:**
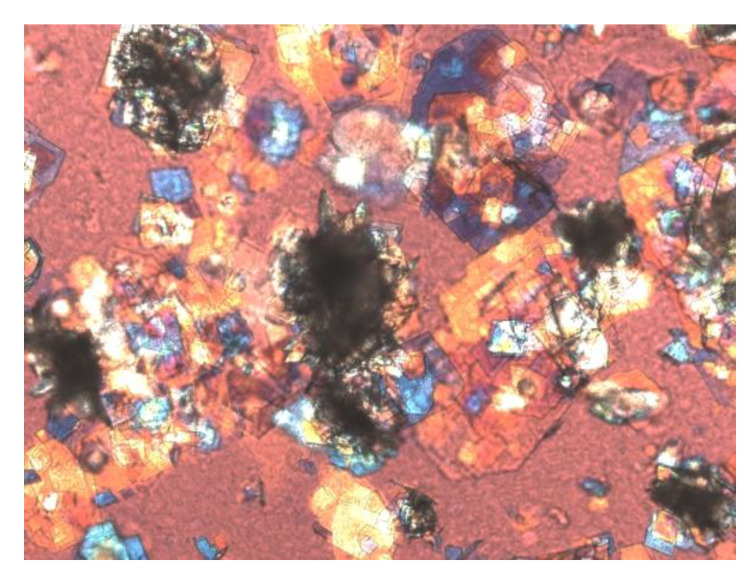
Knockout of the cholecystokinin A receptor (*CCKAR*) gene in mice impairs gallbladder emptying, causing gallbladder stasis. These abnormalities greatly accelerate cholesterol nucleation and crystallization and promote the formation of biliary sludge, i.e., the precursor of gallstones. As studied by phase contrast and polarizing light microscopy, biliary sludge is found in fresh mouse gallbladder bile that consists primarily of classical solid plate-like cholesterol monohydrate crystals embedded in mucin gel or bound by mucin gel to form the aggregates of solid cholesterol crystals. Magnification is 800× by polarizing light microscopy.

As revealed from clinical studies and physical–chemical experiments, gallbladder stasis promotes cholesterol nucleation and crystallization, agglomeration of solid plate-like cholesterol crystals, and stone growth in mucin gel, ultimately forming biliary sludge [63]. Figure 3 shows biliary sludge in fresh mouse gallbladder bile as found by polarizing light microscopy. In turn, biliary sludge can work as a vital nidus for accelerating cholesterol crystallization and the precipitation of calcium bilirubinate [71]. In addition, the viscous mucin gel could mechanically obstruct the cystic duct, further worsening gallbladder emptying.

## 7. Effect of *LITH13* on the Pathogenesis and Pathophysiology of Cholesterol Gallstone Formation in Humans

Based on the finding that the *CCKAR* gene is *Lith13* located on chromosome 5 in mice (Figure 1), it has been revealed that genetic variations in the human *CCKAR* gene are an important risk factor for gallstone formation [34]. It is highly likely that an aberrant splicing of *CCKAR* could cause a nonfunctional receptor activity in a small number of obese patients with cholesterol gallstones [32,33]. However, it is still unclear whether there are mutations or polymorphisms in the *CCKAR* gene in nonobese patients with cholesterol gallstones.

Based on the results from genetic analysis of *Lith13* and pathophysiological studies of gallbladder and gallstone phenotypes in mice, it has been suggested that there is a possible relationship between the abnormality of the *CCKAR* gene and cholesterol gallstone formation in humans. Moreover, abnormal processing of the *CCKAR* gene has been found in obese patients with cholesterol gallstones [32]. However, no mutations or sequence variants in the *CCKAR* gene are revealed in patients with the same phenotypes who received cholecystectomy [33]. Thus, it is still unclear whether variants in the *CCKAR* gene are associated with increased gallstone prevalence in humans and whether there are mutations in the *CCKAR* gene in gallstone patients. In addition, a comprehensive investigation is strongly needed to examine whether single nucleotide variants (SNVs) of the *CCKAR* gene play a role in increasing predisposition to the formation of biliary sludge and gallstones in pregnant women and in patients receiving TPN.

Under normal physiological conditions, different absorption rates of biliary cholesterol, bile salts, and phospholipids by several lipid transporters on the apical membrane of the epithelial cells of gallbladder could diminish cholesterol saturation of bile [25]. Notably, in the lithogenic state, the gallbladder epithelial cells could fail to selectively absorb biliary lipids in patients with cholesterol gallstones. Abnormalities in lipid absorption by the epithelial cells of gallbladder could enhance cholelithogenesis because of persistent supersaturation with cholesterol during the period of bile being concentrated for storage in the gallbladder [145,146,147]. It has been found that in gallstone patients, gallbladder hypomotility still exists even in the stone-free gallbladder after successful extracorporeal shock-wave lithotripsy and dissolution therapy with oral ursodeoxycholic acid (UDCA) [148,149,150]. The degree of impairment of gallbladder emptying is augmented in proportion to the cholesterol concentrations of gallbladder bile, i.e., increased CSI values, even in healthy control subjects. Additionally, diminished interdigestive gallbladder emptying and filling can lead to sustained passage of lithogenic hepatic bile from the liver directly into the duodenum, thus enhancing the effects of the enterohepatic circulation and of the hydrophobicity of bile salt pool on cholesterol crystallization [13,151]. Evidently, patients with gallbladder hypomotility suffer from impaired gallbladder emptying and refilling, and excess amounts of cholesterol accumulated in the gallbladder smooth muscles could work as a myotoxic agent for triggering this event in the gallbladder.

Sluggish gallbladder motility is often uncovered by abdominal ultrasonography in women during pregnancy and oral contraceptive administration, as well as in patients with long-standing total parenteral nutrition (TPN), implying that it is an important risk factor for gallstone formation [152,153,154,155]. Approximately half of patients with Crohn’s disease develop gallstones after receiving TPN, whereas only one fourth of patients with Crohn’s disease and without TPN suffer from gallstones. Throughout the period of TPN, there is an incomplete gallbladder emptying mostly due to a deficit of the stimulus from consumption of meals for triggering CCK secretion. As a result, these abnormalities cause gallbladder stasis, bile stagnation, and the formation of biliary sludge, thus promoting cholesterol crystallization and gallstone formation [156,157,158]. By contrast, repeated gallbladder contraction and emptying as stimulated by supra-physiological doses of CCK-8 could improve gallbladder motility by restoring gallbladder contractility and subsequently prevent the formation of biliary sludge and gallstones in patients receiving TPN [159]. Obviously, daily intravenous infusion of CCK-8 can enhance complete gallbladder emptying, thus eliminating the inevitable risk of biliary sludge and gallstone formation [159]. Therefore, the potent gallbladder-specific CCKAR-selective agonist is likely to offer a new strategy for preventing gallstone formation by increasing gallbladder emptying in women during pregnancy and in individuals with impaired gallbladder contractility.

Celiac disease is a serious, chronic autoimmune disease that occurs in genetically predisposed people in whom the ingestion of dietary gluten leads to damage in the small intestine. The proximal small intestine is the major site of damage with villus atrophy of the mucosa [160]. Accumulated evidence from clinical studies has revealed that during the postprandial period, gallbladder contraction and emptying in response to a fatty meal is dramatically diminished in untreated celiac patients due to a significant defect in CCK secretion by the atrophic small intestinal mucosa, as revealed by a strikingly low concentration of CCK in plasma and small intestinal extracts [17,18,19,20,161,162]. This is because enteropathy can impair CCK release from the small intestine in patients with celiac disease before the gluten-free diet is given [17]. Furthermore, even in the fasting state, the concentration of CCK in circulation is significantly lower in untreated celiac patients compared with healthy control subjects [17]. This indicates that a high-fat diet cannot effectively trigger CCK release by the enteroendocrine I cells in patients with celiac disease because of severe immune-mediated enteropathy including villus atrophy, enterocyte disarray, crypt hyperplasia, epithelial cell layer, and intense inflammation of the lamina propria [163]. As a result, gallbladder stasis is often found in celiac patients before a gluten-free diet is commenced [23], which is a critical risk factor for causing the formation of biliary sludge and gallstones. Additionally, clinical investigations have found that gallbladder responsiveness to CCK could also be impaired [21]. This suggests that there may be a reduced expression level of *CCKAR* in the gallbladder. Another possible explanation is that the binding capability of CCKAR to CCK-8 is disrupted. As dramatic reduction in intestinal CCK secretion could lead to dysfunctional small intestine motility, this could increase intestinal cholesterol absorption and promote the accumulation of excess cholesterol in the gallbladder well, which may lead to dysfunctional CCKAR in the gallbladder. Therefore, celiac disease is a critical risk factor for gallstone formation [24]. The gluten-free diet should start as early as possible so that impaired gallbladder and small intestine motility, as well as gallbladder stasis and biliary sludge can be prevented.

**Figure 4:**
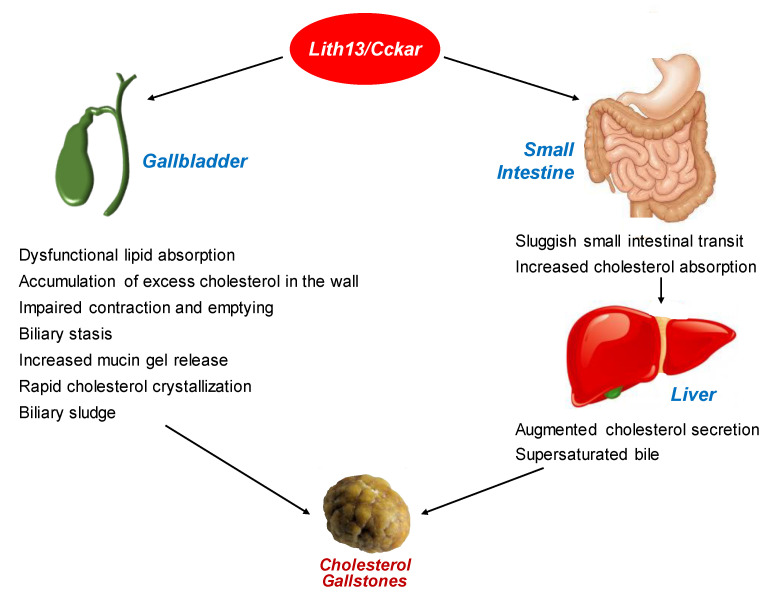
Working model for the critical role of *Lith13/CCKAR* in enhancing cholelithogenesis (see text for further description).

Figure 4 illustrates the lithogenic mechanisms of *Lith13/CCKAR* that works mainly on the gallbladder and small intestine for promoting the formation of cholesterol gallstones.

## 8. Conclusions and Future Directions

Compelling evidence has strongly suggested that the pathogenesis of cholesterol gallstone formation is determined not only by *Lith* genes, but also by hepatic hypersecretion of biliary cholesterol, supersaturated gallbladder bile, rapid cholesterol nucleation and crystallization through several intermediate steps, dysfunctional gallbladder motility, and increased intestinal cholesterol absorption with a dramatic change in gut microbiota [3]. The new concept is that both genetic and environmental factors work together on the complex pathophysiological mechanisms involved in the pathogenesis of cholesterol gallstone formation [164]. Recent progress in understanding the molecular, genetic, and physical–chemical basis of *Lith13* indicates that dysfunctional *CCKAR* enhances susceptibility to cholesterol gallstones through two principal mechanisms: (i) impaired gallbladder emptying plays a key role in promoting the development of bile stagnation, as well as the formation of biliary sludge, i.e., the precursor of gallstones, and microlithiasis; (ii) delayed small intestinal transit augments cholesterol absorption for enhancing biliary cholesterol hypersecretion and increasing the accumulation of excess cholesterol in the gallbladder wall that further exacerbates impaired gallbladder emptying [165]. If these two defects in the gallbladder and small intestine could be prevented by oral administration of the potent CCKAR agonists, the risk of developing cholesterol gallstones could be dramatically reduced, particularly for women during pregnancy, patients receiving TPN, and subjects with gallbladder contractile dysfunction, e.g., patients with celiac disease.
